# The Effect of an Automated Phone Warning and Health Advisory System on Adaptation to High Heat Episodes and Health Services Use in Vulnerable Groups—Evidence from a Randomized Controlled Study

**DOI:** 10.3390/ijerph15081581

**Published:** 2018-07-25

**Authors:** Kaddour Mehiriz, Pierre Gosselin, Isabelle Tardif, Marc-André Lemieux

**Affiliations:** 1 Doha Institute for Graduate Studies, School of Public Administration and Development Economics, P.O. Box: 200592, Zone 70, Al Tarfa Street Al-Daayen, Doha, Qatar; 2 Institut National de la Santé Publique and Ouranos, 945 Avenue Wolfe, Québec, QC G1V 5B3, Canada; Pierre.gosselin@inspq.qc.ca; 3 Direction de Santé Publique de la Montérégie, 1255 rue Beauregard, Longueuil, QC J4K 2M3, Canada; Isabelle.tardif.agence16@ssss.gouv.qc.ca (T.I.); marc-andre.lemieux.agence16@ssss.gouv.qc.ca (M.A.-L.)

**Keywords:** automated phone warning systems, heat waves warnings, public health, impact evaluation, randomized controlled trial design, climate change adaptation

## Abstract

Automated phone warning systems are increasingly used by public health authorities to protect the population from the adverse effects of extreme heat but little is known about their performance. To fill this gap, this article reports the result of a study on the impact of an automated phone heat warning system on adaptation behaviours and health services use. A sample of 1328 individuals vulnerable to heat was constituted for this purpose and participants were randomly assigned to treatment and control groups. The day before a heat episode, a phone heat warning was sent to the treatment group. Data were obtained through two surveys before and one survey after the heat warning issuance. The results show that members of the treatment group were more aware of how to protect themselves from heat and more likely to adopt the recommended behaviours. Moreover, a much smaller proportion of women in this group used the health-care system compared to the control group. Thus, the exposure to an automated phone warning seems to improve the adaptation to heat and reduce the use of health services by some important at-risk groups. This method can thus be used to complement public health interventions aimed at reducing heat-related health risks.

## 1. Introduction

Climate change is assumed to lead to an increase in the frequency and intensity of heat waves [1]. Heat waves are an important public health issue as they lead to increased mortality, hospitalizations, and morbidity [2,3,4,5,6]. The elderly and chronically ill are particularly vulnerable to this weather hazard [6,7], and their vulnerability is accentuated by certain housing and neighbourhood characteristics such as the unavailability of residential air conditioning and lack of green space in the surrounding environment [8,9]. In addition to health concerns, heat waves have also negative impacts on different aspects of the ecological and social systems such as an increase in energy consumption, bushfire occurrences, and crop damage risks, as well as the degradation of water quality and availability [10].

Warning systems are among the main ways to fight extreme weather events, including heat waves [11,12,13]. Heat wave warnings are typically issued when the temperature reaches levels posing a significant risk to the population [12,14]. However, as many studies show, hot temperatures well below these thresholds are dangerous for the elderly or chronically ill individuals given their reduced physiological adaptation capacity to heat [15,16,17], as well as for low income individuals or persons suffering from mental health issues or social isolation [12,18]. These studies thus suggest that the thresholds generally used by public warning systems are not adapted to the needs of the most heat-wave vulnerable persons.

In addition to questions on the appropriate choice of warning thresholds, concerns have arisen about the ability of public warning systems to effectively reach people vulnerable to heat. Indeed, a significant percentage of the population is unaware of heat warnings and, therefore, unprepared to protect themselves in such an event [19,20]. This means that a portion of the population is underserved by traditional warning systems relying on public media such as television and radio.

Automated phone warning systems, in addition to announcing high-risk heat periods, can also be used to inform and provide specific health advice when the temperature rises to levels considered hazardous for the most vulnerable groups, yet are lower than the fixed thresholds for issuing general heat wave warnings. By targeting only the most vulnerable groups, automated phone warning systems avoid the multiplication of warnings in the media and the resulting risk of decreased performance due to warning fatigue, as documented in Australia [21]. Furthermore, they cost less because they require using fewer human resources than phone calls by health care workers or home visits by police officers and firemen, the three categories of professionals typically used in Quebec—Canada to contact certain categories of at-risk people during a heat wave. This technology thus holds the promise of making available, at a reasonable cost, a warning and advisory service that is more adapted to the needs of people most at risk.

Studies on the performance of heat warning systems are limited, but the available evidence suggests that warnings improve heat protection knowledge and behaviours [20,22,23,24] and reduce the number of hospitalizations and the risk of death [25,26,27,28]. Although these studies have significantly contributed to the development of knowledge on this issue, in most cases the methodology used cannot effectively control confounding factors that may bias the calculation of outcomes [19,29]. Nor do these studies specifically focus on the impacts of automated warning systems, although the use of this technology is increasing.

The purpose of this study, then, is to measure the effects of an automated phone warning and advisory system on people vulnerable to heat using an experimental design, which constitutes an original contribution to research on the performance of warning systems during heat episodes.

## 2. Material and Method

### 2.1. Description of the Intervention

The automated phone warning and advisory system, Téléphone Santé, is a pilot project designed to contact heat-vulnerable people to warn them of heat episodes and provide recommendations about protecting their health in such circumstances. The system was tested on a sample of residents in the Longueuil area, on the south shore of Montreal (Canada), presenting certain pre-existing vulnerabilities described below.

The warning system was programmed to issue heat warnings and health advice when the maximum temperature was forecast to exceed 31 °C (88 °F) for one day or more. This threshold was selected because, on the one hand, temperatures higher than or equal to 31 °C are a significant cause of death for people vulnerable to heat in the study area [30]. On the other hand, setting the threshold at higher level reduces considerably the probability of testing the system within the timeframe of this study as the frequency of heat episodes with temperatures exceeding 31 °C in the Longueuil area is low.

The programmed warning message was about 66 s long, announced the forecast high temperature and provided the following tips, which are generally recommended to help people protect themselves from heat [12]: avoid physical activity; drink plenty of water; use a fan or an air conditioner; take cool showers and baths; spend time in cool or air-conditioned places; stay in the shade when outdoors and wear light clothing; in an emergency, call 911 (emergency services); for health-related questions call 811 (24/7 health information services). The system was also programmed to send a reminder on the second day of the heat episode stressing the importance of some preventive tips. A public health physician was heard in all the messages.

Between 16 and 20 August 2015, the Longueuil area had a heat episode during which the maximum temperature repeatedly reached the warning-triggering threshold. It was also very humid during this time. On 15 August 2015, the day before the onset of the heat episode, members of the experimental group received a phone heat warning from Téléphone Santé along with the public health advice listed above. The purpose of this study is to measure the effects of exposure to this phone warning.

### 2.2. Methodology

#### 2.2.1. Outcome Measurement Variables

Exposure to heat warnings is meant to improve recipients’ knowledge of heat episode occurrences and heat protection measures [20,31], motivate them to adopt the recommended behaviours [19,20,23,24,32], reduce heat-related health risks and, ultimately, reduce health and social services use [28,33,34,35]. These expected outcomes were therefore used to evaluate the effects of Téléphone Santé.

▪ Improving awareness of heat episodes and heat protection measures:

Data on the effects of heat warnings were obtained through surveys of study participants. Two questions were used to determine whether or not respondents were informed of the heat episode. The first question determined whether the respondent was aware of the heat episode; the second, the date on which the respondent became aware of it, i.e., before, during or after the heat episode.

As for awareness of recommended heat protection measures, respondents had to answer an open-ended question on the best ways of protecting themselves from heat. The number of measures cited by each respondent that matched the recommendations in the warning message was then calculated. This question could be viewed as assessing the persuasiveness of the warning message. Thus any change in the response due to the exposure to heat warning messages can be interpreted as an indication of the message’s capacity to influence the respondents’ beliefs about the best ways to protect themselves from heat.

▪ Adopting recommended heat protection behaviours:

The effects of the warnings on the behaviours mentioned in the warning message were measured. These behaviours included drinking water, using fans and air conditioners, taking cool showers and baths, spending time in cool or air conditioned places, staying in the shade when outdoors, and reducing physical activity.

For drinking water and reducing physical activity, the participants had to rate, on a scale of 1 to 5, whether they did this more or less than usual during the heat episode. The same scale was used for assessing the extent to which people who went outside during the heat episode protected themselves by staying in the shade. For the other variables, the respondents were asked to indicate whether they followed the advice (yes/no) and, if yes, to indicate on a scale of 1 to 5 if they did so more or less than usual.

▪ Reducing heat-related illnesses:

Respondents had to indicate whether, during the heat episode or the following two or three days, they experienced headaches, muscle cramps, chest pain, breathlessness, unusual fatigue or any other symptoms that are potentially related to excessive heat. A dichotomous variable was then created based on these categories, indicating whether or not the respondent experienced any of these symptoms.

▪ Reducing health services use:

Six questions were asked to determine whether, during the heat episode or the following two or three days, respondents: (1) called a nurse, pharmacist or doctor; (2) called 811 (Info Santé); (3) were hospitalized; (4) visited an emergency room; or (5) consulted a doctor or nurse at a clinic; or (6) consulted a pharmacist. A dichotomous variable was then created based on these categories, indicating whether or not the respondent used at least one of these services.

#### 2.2.2. Recruiting Participants and Assigning Groups

Participants were recruited by the Montérégie public health authority (Direction de santé publique de la Montérégie) in conjunction with the municipalities and community organizations of the City of Longueuil. The call for participation in this experiment was published in local newspapers, websites and social media, and advertised in municipal buildings. The community organizations and the Public Housing Office scheduled meetings with their members at which the researchers presented the research project and the conditions for participation. Potential participants could register in person at these meetings, or later by phoning or emailing the project leaders, or online. A total of 1328 individuals meeting the eligibility criteria signed up for the project; 76% were women. Participants had to meet at least one of the following heat and smog vulnerability criteria: be 65 years of age or older, present a heart or lung medical condition, suffer from diabetes, kidney failure or a neurological disorder, or, lastly, have mental health issues. All registrants signed a research participation consent form as per the Ethics certification requirements.

Ethics certification: The protocol of this study was approved by the ethics committee of the Institut national de la recherche scientifique: CER-15-370.

Participants were randomly assigned to the treatment group and control group. The random assignment was based on telephone numbers to preclude the risk of intergroup contamination. People with the same phone number were thus randomly assigned to the experimental or control group using STATA software. At the end of the process, there were 662 people in the first group and 666 in the second.

#### 2.2.3. Data Collection

The study questionnaire was prepared by the authors based on questionnaires previously used in similar studies [2,20]. It was then reviewed by five public health experts and tested on 22 people similar to the clientele targeted by Téléphone Santé. The final version was also pretested by the survey firm to check the items for clarity.

Data were collected through three surveys. The first survey took place immediately after the participants were recruited, from 25 June to 14 July 2015, and was intended to gather data on the participants’ socioeconomic and demographic characteristics, as well as on the quality of the recruiting process. The response rate was 79.4%. The second survey was administered after the first heat episode from 27 July to 29 July 2015, during which no automated heat warning messages were issued by the system. This survey targeted respondents to the initial survey and was intended to collect data on the outcome measurement variables of Téléphone Santé in the absence of warning messages (baseline data). The response rate was 82.6%.

**Figure 1:**
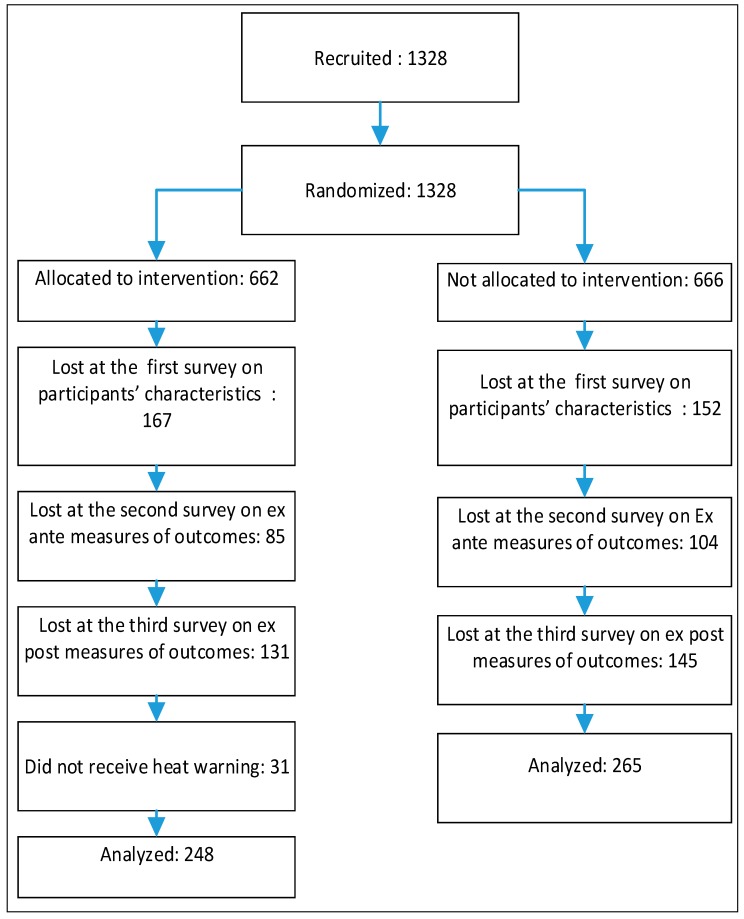
CONSORT Flow Chart of the Experiment.

The day before the heat episode of 16–19 August 2015, a warning message was sent to members of the treatment group. Immediately after the heat warning was lifted, the study participants were surveyed in order to obtain information on the outcomes of the warning message. A response rate of 72.3% was obtained. Figure 1 provides more information on the participant recruiting process, formation of groups and, lastly, data collection and analysis.

Although these sample response rates were generally very satisfying, the three waves of the survey resulted in a significant reduction of the sample size. In addition to the loss of statistical power, sample attrition raises the risk of engendering differences between the treatment and control groups. This risk is minimized in this study because respondents did not know if they belonged to the treatment or control group when they responded to the first two surveys and, thus, their decision to respond to these surveys or not were not correlated with their experimental/control status in the project. Likewise, in all three surveys, the response rate of the two groups was the same, as shown in the Consolidated Standards Of Reporting Trials (CONSORT) flow chart (Figure 1). Last, data on admissibility criteria collected at the moment of the participants’ enrollment indicate that, for each survey, there are no statistically significant differences between the treatment and control groups.

#### 2.2.4. Data Analysis

In order to measure the differences between the treatment and control groups, we used two-tailed *t*-tests as the means for independent samples for continuous variables, two-sided Z-tests of proportions for independent samples for binary variables and Wilcoxon-Mann Whitney test for independent samples for ordinary variables.

As indicated in the Section 3.3 on baseline differences, only three of the 17 outcome variables have statistically significant differences between treatment and control groups (*p* < 0.10). Difference in differences estimator was then used for those three variables to adjust for these differences [36]. This design was implemented by using the following two-period panel model [37,38]:

(1) $$\mathrm{Oit}\;=\;\alpha \;+\;\beta_{1}\mathrm{Ti}\;+\;\beta_{2}\mathrm{Vt}\;+\;\beta_{3}\mathrm{Wit}\;+\;\mathrm{Eit}$$

Oit: Outcome variable for the individual i during the period t

α: An intercept

Ti: Group membership of the individual i (Treatment group = 1 and control group = 0)

Vt: Heat episode (t = 1 or 2)

Wit: individual i was exposed to heat warning (yes = 1, no = 0)

Eit: Error term.

In this model, the coefficient β_3_ of the variable Wit measures the impact of heat warning.

## 3. Results

### 3.1. Sample Characteristics

Table 1 presents the data on sample characteristics obtained from the initial survey (before any intervention). Data on admissibility criteria indicate that 87% of the participants were 65 years of age or older and 69% had chronic illnesses related to heat vulnerability. Among the latter, 52% suffered from cardiovascular diseases, 22.6% from bronchial and lung diseases, and 19.7% from diabetes. Kidney diseases and neurological problems were less prevalent with 5.9% and 7.4% of the respondents having these conditions, respectively. The two tailed Z-tests of proportions presented in Table 1 indicate that there are no significant statistical differences between the treatment and control groups regarding these variables.

With regard to the variables that are not related to admissibility criteria, 75% of the respondents were women and 53% had a university degree or college diploma with no differences between treatment and control groups. Data on income also indicate that the two groups are equivalent except for the category of people earning $25,000 or less where the members of the treatment group are overrepresented. The analysis of the differences between treatment and control groups thus lend support to the idea that the randomization process was well implemented.

### 3.2. The Quality of the Heat Episode Warning

The impacts of weather warning systems depend to a large extent on the quality of warning messages such as the reliability of forecasts and utility of the advice provided [20,39].

Three dimensions were used to measure the quality of the heat episode warning issued by Téléphone Santé. These are the reliability and usefulness of the heat warning message and the ease of understanding it. Respondents were also asked to assess their overall level of satisfaction with the warning message. Each item was measured by a five-level scale (1 corresponding to the highest level and 5 to the lowest level), except for the reliability of the message that was scored on a four-level scale. 

Data on the message’s quality were obtained from the treatment group as part of the third survey on the effects of the exposure to heat episode warning. Table 2 indicates that the treatment group had a very positive evaluation of the quality of warning. All respondents considered that the heat warning was reliable (78.3% + 21.7%) and most of them found the message useful (66.6% + 28.5%) and easy to understand (75.2% + 20.2%). Furthermore, 95.7% were satisfied with the quality of the warning message (70.6% + 25.13%).

### 3.3. Baseline Differences

Table 3 presents the data on the first heat episode, during which no warning was issued by Téléphone Santé. To verify the ex-ante equivalence between the two groups, only data on people who responded to the two heat episode surveys were reported in this table. A total of 31 members of the treatment group did not receive heat warnings and, given that they were not exposed to the treatment, were not included in the analysis in order to improve the internal validity of the study. 

Average scores are reported for ordinal variables and percentages for binary variables. Data on outcome variables show no statistically significant differences at the 5% risk of error level between the two groups except in the case of the frequency of using air conditioner (4.28 versus 4.10, *p* = 0.03). Using a 10% risk of error, we also observe differences between the two groups concerning heat-related illness (56% versus 48%, *p* = 0.08) and frequency of using fans (4.31 versus 4.13, *p* = 0.08). The difference in the difference estimator was used to adjust for these baseline differences.

In the case of certain variables, the number of observations is significantly lower than the average. However, this is not due to high levels of missing data but simply to the use of filter questions for some outcome measures. For instance, only for those using fans during a heat episode (86.1%) was an additional question on the frequency of use asked.

### 3.4. Effects of Automated Phone Warnings

Table 4 presents the results relating to the variables showing no statistically significant differences (*p*-value > 0.10) between treatment and control groups regarding baseline measures.

#### 3.4.1. Effects on Awareness of Heat Episode and Heat Protective Behaviours

The data of Table 4 suggest that almost all respondents (98%) knew that temperatures in their area had been equal to or above 31 °C between 16 and 19 August 2015, and show no significant difference between treatment and control groups in this respect. Furthermore, 75.6% of the members of the experimental group were informed of this heat episode prior to its onset versus 68% of the control group, but this difference is not statistically significant (*p* = 0.08). The warning thus appears to have had no effect on whether the participants were informed of the occurrence of the heat episode. Members of the experimental group, however, were slightly more likely to mention recommended behaviours among the best protection from heat episodes, citing 2.3 of the 7 recommended measures on average versus 2.1 for the control group (*p* = 0.02).

#### 3.4.2. Effects on the Adoption of Recommended Protective Behaviours

Data on protective behaviours during heat episodes indicate that the treatment group is more likely to increase its consumption of drinking water than the control group (4.19 versus 4.04, *p* = 0.02) and to take cool showers or baths (81.4% versus 73.6%, *p* = 0.04). In addition, members of the experimental group were less likely to go outside during the heat episode than those in the control group (62% versus 70.3%, *p* = 0.05).

The data also show that an equivalent percentage of the treatment and control group spent time in cool or air conditioned places (38.6%). However, the treatment group registered a high score regarding the frequency of this behaviour (3.57 versus 3.28, *p* = 0.00). For the other protective behaviours listed in Table 4, they show only insignificant differences.

For the variables frequency of using fans and frequency of using air conditioner, a panel ordinal regression model was used to control for statistically significant baseline differences, as mentioned in the methodology section. A panel logit model was used for the same purpose in the case of the risk of suffering from heat related symptoms variable. The results of this model are presented in Table 5. They suggest that after adjusting for pre-existing differences, exposure to heat warnings has no effect on the frequency of using fans and air conditioning.

The analysis of the effects on behaviours suggests that heat episode warnings motivate people to stay indoors when it is hot, drink more water, spend more time in cool and air conditioned places and take cool baths and showers. On the other hand, the warnings apparently had no effect on reducing physical activity, staying in the shade or using air conditioners and fans.

#### 3.4.3. Health Outcomes

Two indicators were used to measure the health outcomes of heat episode warnings: the percentage of people who had heat-related symptoms and the percentage of people who used the health care system.

Table 5 indicates that after adjusting for baseline differences, there were no significant differences between the treatment and control groups regarding suffering from heat-related symptoms (OR = 1.18, *p* = 0.60). The analysis of the effects of the warnings on health services use presented in Table 4 shows that 7.7% of the members of the treatment group used the health care system versus 9.4% of the control group, but this difference is not statistically significant (*p* = 0.48). However, women in the experimental group used the health care system less than women in the control group (5.7% versus 11.3%, *p* = 0.05). This difference between the two groups is significantly greater in the case of women with chronic illnesses (6.2% versus 13.5%, *p* = 0.02), a 54% difference. The analysis thus suggests that automated phone heat warnings reduce health services use by women, particularly those with chronic illnesses related to heat vulnerability.

## 4. Discussion

The study results suggest that automated phone warnings improve people’s awareness of heat protection measures. Warnings with recommendations also made people more likely to follow the heat-related advice to stay well hydrated, remain indoors, take cool showers and baths, and spend time in cool or air conditioned places, but had no effect on the other recommended behaviours. Overall, in terms of health outcomes, heat warnings do not appear to reduce the percentage of people presenting heat-related symptoms or using the health care system. However, there was a significant reduction of health services use by women, especially those with chronic illnesses.

As is the case of some studies [40,41], we find that women are particularly vulnerable to heat as 56% of them reported suffering from heat-related illnesses versus 41% of the men. These sex differences have been linked to hormonal physiology, and differences in skin blood flow, sudation and thermosensitivity [42,43]. Given that women account for about half of the population and even more in older strata and the prevalence of chronic illnesses is increasing as the population ages [44], automated phone warning systems seem to help protect a large group of people who are among the most vulnerable to heat and significantly reduce their health services use.

This finding is important in the current context of climate change, with public health authorities having to innovate and diversify their interventions in order to better protect a population with heterogeneous needs and levels of vulnerabilities from an increasing risk of heat waves [45]. In particular, this study suggests that automated phone warning systems with specific guidelines help improve the knowledge and behaviours of the most vulnerable people to heat and relieve pressure on the health care system during heat episodes. Two Canadian studies noted that medical consultations during or after heat waves concerned between 10% and 12% of the adults [8,46]. These systems can therefore be a useful component of heat wave emergency plans. The low cost of automated phone warning systems makes it possible to use them for adapting heat warning services to specific needs of people who, due to age or chronic health issues, are sensitive to temperatures well below the thresholds typically used to trigger public heat wave warnings. However, implementing automated phone warning systems requires identifying and recruiting vulnerable people, continually updating these lists, and lastly, a close cooperation between weather forecasting services and the public health authorities in charge of issuing heat warnings.

This study has at least three limitations that are worth noting. First, the use of surveys involves the risk that, due to social desirability concerns, respondents may be induced to misreport their true behaviour during the heat episodes. Secondly, information provided to the participants on the objectives of the project could improve their heat-related knowledge and behaviour. However, the use of experimental and control groups, as is the case in this study, permits neutralisation of this potential threat to internal validity, as the two groups are largely exposed to the same social pressures and information on the project. Lastly, the external validity of this study may not be as strong as expected because it concerns only two episodes of heat in a given city, which reduces the possible generalization of the conclusions to other geographic areas and periods. In addition, participants were not randomly selected from the general population, given the research context and need for informed consent to participate in this study. And, even if random selection was used, the simple fact of taking part of an experiment may motivate participants to actively seek information on the risks of heat waves as well as on the protective behaviours. They may thus display some characteristics that differentiate them from the general population and, consequently, the impacts of Télephone Santé on the population would not be the same as those reported in this study. However, as is the case of impact evaluations using experimental designs, the strong capacity to deal with the threats to internal validity come at the price of a limited possibility to generalize study results to other contexts [47]. The replication of this experiment in different contexts would thus improve the external validity of the findings.

## 5. Conclusions

Automated phone warning systems appear to be a promising way to improve vulnerable peoples’ level of preparedness for heat episodes. However, it should be noted that the aim of this study was not to compare the automated phone warning system to conventional warning systems using the public media at large, but simply to test whether this low-cost method is effective for high-risk subgroups. It should thus not be inferred that this system is an alternative to the current warning methods by meteorological services, but rather a complement that can be parameterized and used to target subpopulations that are more vulnerable to heat, make the risks appear more personal, and as well remind the usual preventive actions for users. Such subgroups may not be well protected by the current systems using higher threshold levels for heat warnings.

## Figures and Tables

**Table 1:** Participant characteristics.

Variable	Treatment		Control		*p* Value
	N	%	N	%	
Cardiovascular disease	492	51.0	508	52.9	0.54
Bronchial or lung disease	491	22.6	507	22.5	0.96
Diabetes	489	19.4	509	20.0	0.81
Kidney failure	488	4.9	509	4.9	1.00
Neurological diseases	487	7.8	510	7.3	0.84
65 years or over	495	87.1	511	86.3	0.87
Women	479	74.5	500	75.8	0.65
Elementary school diploma	487	15.6	502	12.2	0.12
Secondary school diploma	487	32.9	502	34.1	0.69
College diploma	487	20.9	502	19.7	0.63
University degree	487	30.6	502	34.1	0.24
Household income (Thousands $): Less than 25 Between 25 and 50 Between 51 and 75 More than 75	395	38.1	394	31.1	0.04
	395	33.3	394	36.2	0.38
	395	17.5	394	18.5	0.72
	395	11.2	394	14.2	0.20

N: Number of respondents to each question.

**Table 2:** The quality of heat episode warning message.

	1	2	3	4	5
Reliability of the heat warning (1: very reliable, 4: very unreliable)	78.3%	21.7%	0%	0%	NA
Usefulness of the heat warning (1: totally useful, 5: totally useless)	66.6%	28.5%	2.7%	0.5%	1.6%
Ease of understanding the heat warning message (1: very easy, 5: very difficult)	75.2%	20.2%	1.6%	0.8%	2.2%
Level of satisfaction of the heat warning (1: very satisfied, 5: very dissatisfied)	70.6%	25.13%	2.4%	1.1%	0.8%

NA: Not applicable because a four-level scale was used.

**Table 3:** Ex ante comparison between treatment and control groups on outcome variables.

Outcomes	Treatment Group		Control Group		*p* Value
	N	Mean or %	N	Mean or %	
Were informed of the heat episode	247	96.3%	262	95.4%	0.60
Were informed of the heat episode before its onset	216	59.3%	225	56.4%	0.55
Average number of recommended heat protection measures cited (a *t*-test was used)	247	2.2	265	2.2	1.00
Frequency of drinking water	245	4.08	260	4.01	0.27
Frequency of physical effort	238	3.93	259	3.91	0.87
Took cool showers or baths	245	75.6%	260	69.6%	0.20
Frequency of taking cool showers or baths more than usual	181	3.52	180	3.49	0.47
Visited cool or air-conditioned places	246	45.5%	259	38.6%	0.12
Went outside during heat episode	242	69.8%	258	72.4%	0.51
Frequency of spending time in cool or air-conditioned places	110	3.25	100	3.18	0.29
Frequency of staying in the shade	160	3.83	179	3.78	0.56
Used fans	180	86.1%	173	89.0%	0.41
Frequency of using fans	154	4.31	152	4.13	0.08
Used air conditioner	193	96.8%	222	96.8%	0.98
Frequency of using air conditioner	185	4.28	214	4.10	0.03
Suffered heat episode related symptoms	246	55.6%	259	47.9%	0.08
Used health care system	246	8.5%	259	9.3%	0.77

**Table 4:** Ex post comparison between treatment and control groups regarding outcome variables.

Outcomes	Treatment Group		Control Group		*p* Value
	N	Mean or %	N	Mean or %	
Were informed of the heat episode	245	98.9%	265	97.4%	0.24
Were informed of the heat episode before its onset	217	75.6%	222	68.0%	0.08
Number of recommended heat protection measures cited (a *t*-test was used)	247	2.32	265	2.07	0.02
Frequency of drinking water	246	4.19	262	4.04	0.02
Frequency of physical effort	242	3.99	261	3.95	0.64
Took cool showers or baths	247	81.4%	265	73.4%	0.04
Frequency of taking cool showers or baths more than usual	200	3.65	195	3.51	0.06
Visited cool or air-conditioned places	246	38.6%	264	38.6%	1.00
Frequency of spending time in cool or air-conditioned places	95	3.57	99	3.18	0.00
Went outside during heat episode	245	62.0%	263	70.3%	0.05
Frequency of staying in the shade	146	3.87	174	3.84	0.65
Used fans	191	0.91	185	0.88	0.44
Used air conditioner	194	97.4%	221	99.1%	0.19
Used health care system	247	7.7%	265	9.4%	0.48
Used health care system (women)	176	5.7%	203	11.3%	0.05
Used health care system (women with chronic illness)	129	6.2%	148	13.5%	0.04

**Table 5:** Estimating the effects of weather warnings using ordinal regressions.

Outcomes	Odds Ratio (Standard Error)	Confidence Interval (95%)	*p* Value
Frequency of using fans	1.38 (0.51)	(0.67–2.84)	0.38
Frequency of using air conditioning	0.67 (0.21)	(0.35–1.23)	0.19
Suffered heat episode related symptoms	1.18 (0.38)	(0.63–2.23)	0.60
